# Hydrothermally Synthesized SnS_2_ Anode Materials with Selectively Tuned Crystallinity

**DOI:** 10.1002/smsc.202400516

**Published:** 2024-12-23

**Authors:** Akzhan Bekzhanov, Nurgul Daniyeva, Qixiang Jiang, Yuri Surace, Freddy Kleitz, Damian Cupid

**Affiliations:** ^1^ Center for Transport Technologies Austrian Institute of Technology GmbH 1210 Vienna Austria; ^2^ Department of Functional Materials and Catalysis, Faculty of Chemistry University of Vienna Währinger Str. 42 1090 Vienna Austria; ^3^ Vienna Doctoral School in Chemistry (DoSChem) University of Vienna Währinger Str. 42 1090 Vienna Austria; ^4^ Core Facilities Nazarbayev University Astana 010000 Kazakhstan; ^5^ Institute of Material Chemistry, Faculty of Chemistry University of Vienna Wähinger Strasse 42 1090 Vienna Austria

**Keywords:** anode, diffusion kinetics, hydrothermal method, semicrystalline SnS_2_, tin disulfides

## Abstract

SnS_2_‐based anode active materials for lithium‐ion battery applications are synthesized with varying degrees of crystallinity via a hydrothermal method, and their electrochemical performance properties are assessed. Different ratios of tin chloride and thioacetamide precursors are used and studied to control the crystallization. In situ electrochemical impedance spectroscopy and galvanostatic intermittent titration technique experiments are used to study the lithium‐ion diffusion kinetics into the crystal structures and the conversion reaction mechanisms for discharge up to *x* ≈ 2.08 moles of lithiation per SnS_2_, equivalent to a discharge capacity of 300 mAh g^−1^. Transmission electron microscopy reveals the presence of amorphous and crystalline domains, as well as the existence of additional Sn_2_S_3_ layers on one of the samples. The highest specific reversible capacity during cycling and rate performance are 598 mAh g^−1^ after 100 cycles and 605 mAh g^−1^ after rate capability test, which are obtained for the samples prepared with the 1:4 tin chloride to thioacetamide ratio.

## Graphical abstract

SnS_2_ is synthesized using different ratios of tin‐to‐sulfur precursors and the electrochemical performance of the samples is investigated. It is found that although the electrode processing results in agglomeration of the particles, the degree of crystallinity of the nanopowders plays a more important role in defining the electrochemical performance of this class of materials.

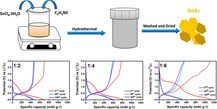

## 1 Introduction

The fast depletion of fossil fuels and climate change associated with their use has led to the need to develop technologies for sustainable energy storage and use. Lithium‐ion batteries (LIBs) are considered as a key technology to ensure a clean transition supporting the use of energy from renewable sources. A great volume of research has thus been performed on developing and understanding the electrochemical performance of electrode materials for LIBs. Electrodes with higher reversible capacity (anode and cathode) operating at sufficiently high voltages (cathode) and delivering good rate capabilities (cathode and anode) are required to improve the energy and power densities of LIBs. Research in anode development for LIBs has mainly focused on incorporating higher contents of silicon into the Si–C composite anode while maintaining stable cyclic performance and high Coulombic efficiencies (CEs). However, conversion anodes based on active materials other than silicon are still of interest, as they would increase the choice of materials and enable flexibility in application‐oriented LIBs design.

**Figure 1:**
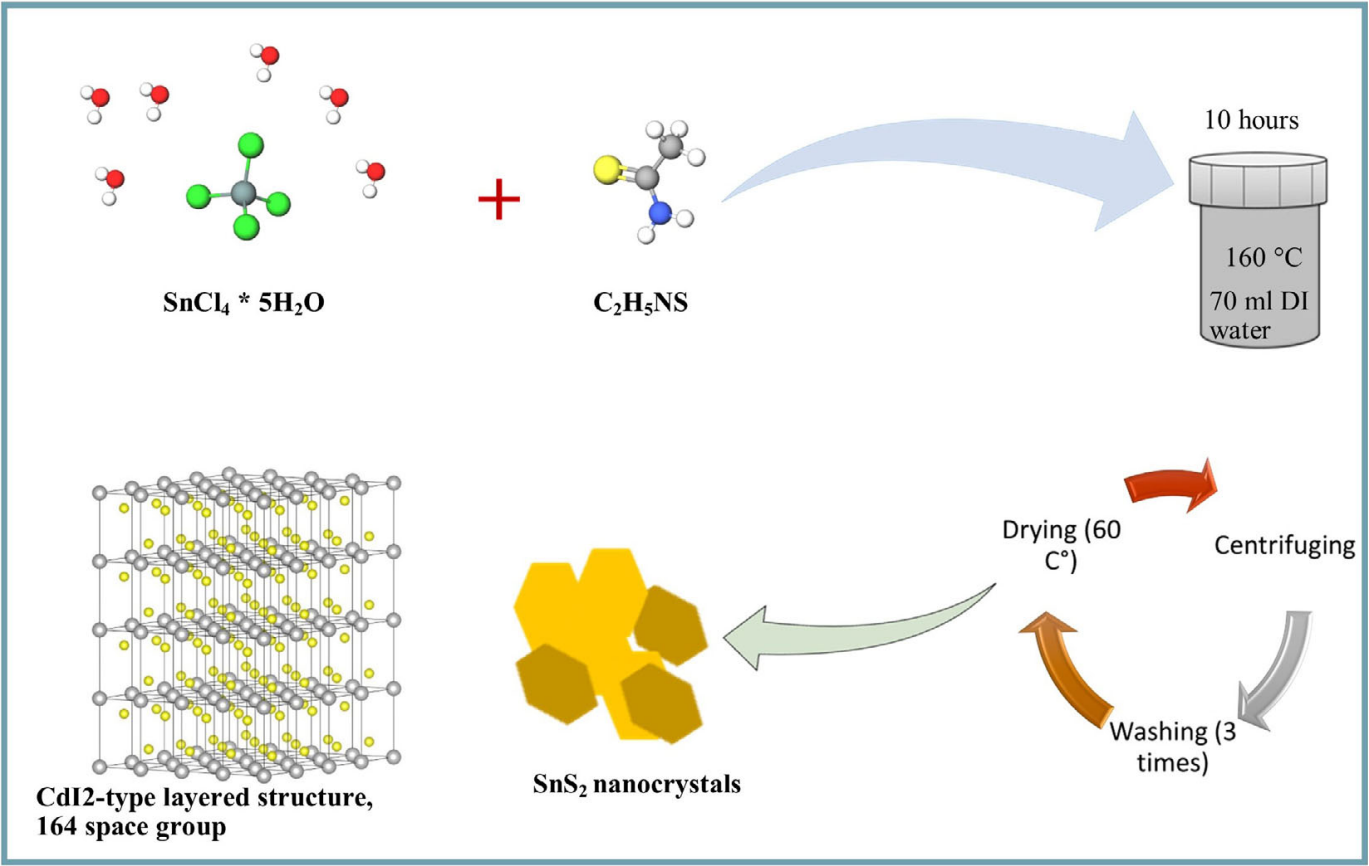
Schematic illustration of the hydrothermal synthesis of SnS_2_ particles.

The SnS_2_ dichalcogenide^[^ ^1^, ^2^, ^3^, ^4^ ^]^ is a promising anode‐active material for LIBs. It exhibits a CdI2‐type layered structure (*a* = *b* ≈ 3.65 Å, *c* ≈ 5.90 Å, 164—space group P3m1) composed of a layer of tin atoms sandwiched between two layers of octahedrally coordinated sulfur atoms, see **Figure** **1**. The intralayer bonding between the tin and sulfur atoms is covalent, whereas the layers are bonded weakly to each other via interlayer Van der Waals interactions along the [001] direction. Due to the preferred growth of the energetically stable [001] plane, the primary particles of SnS_2_ usually adopt a platelet‐like structure. The stacked layers within the platelets allow a fast diffusion of lithium into and out of the particles. SnS_2_ has a high theoretical specific capacity of 1230 mAh g^−1^ based on the transfer of 8.4 electrons per SnS_2_ unit. Electrochemical lithiation of tin disulfide occurs via intercalation, conversion, and alloying reactions.^[^ ^1^ ^]^ These reactions are given below.

(1) $${\mathrm{SnS}}_{2}+x{\text{Li}}^{+}\;+\;xe^{-}\to {\text{ Li}}_{x}{\text{SnS}}_{2\;}(\text{intercalation})$$

(2) $${\text{Li}}_{x}{\text{SnS}}_{2}+(4\;–\;x){\text{ Li}}^{+}+\;(4\;–\;x)e^{-}\to \;2{\text{Li}}_{2}\text{S + Sn }(\text{conversion})$$

(3) $$\text{Sn}\;\text{+}\;{\text{4.4 Li}}^{+}\;+\;4.4e^{-}⇌{\text{Li}}_{17}{\text{Sn}}_{4}\;(\text{alloying})$$

Tailoring the nanomorphology of SnS_2_ has been used to improve its electrochemical performance, with some examples being the use of nanoplates, nanosheets, and hierarchical structures.^[^ ^5^, ^6^, ^7^, ^8^, ^9^ ^]^ **Table** **1** shows available data survey of hydrothermally synthesized SnS_2_ as anode material for LIBs.

**Table 1:** Summary of the precursor ratios and electrochemical testing data for SnS_2_ samples prepared via hydrothermal synthesis.

Synthesis details	Mole ratio of Sn‐ to S‐based precursor	Potential window [V]	Current density [A g^−1^]	Specific capacity [mAh g^−1^]	Number of cycles	References
Na_2_S·9H_2_O solution, SnCl_4_ × 5H_2_O solution, 150 °C for 16 h	1:2	0.005–3.0	0.1	547	50	[45]
tin tetrachloride pentahydrate, thiourea, 180 °C for 20 h	1:5.3	0.05–1.2	0.1	540	30	[9]
SnCl_4_·5H_2_O and l‐cys, 180 °C for 9 h	1:32	0.001–1.3	0.65	570	100	[46]
SnCl_2_·2H_2_O, Na_2_S_2_O_3_·5H_2_O, sulfur powders and hexadecyl trimethyl ammonium bromide, tartaric acid, sodium hydroxide, ethanol, 200 °C for 12 h	1:5:5	0.001–1.2	0.065	532	60	[47]
SnCl_4_·5H_2_O, thiourea, urea, 180 °C for 12 h	1:26	0.005–3.0	0.1	472	100	[48]
SnCl_4_·5H_2_O, thioacetamide, citric acid monohydrate, 2 h under microwave radiation at 180 °C	1:2	0.01–3.0	0.1	327	100	[49]
SnCl_4_·5H_2_O, thioacetamide, 160 °C for 24 h	1:6	0.005–1.1	0.1	515	50	[50]
SnCl_4_·5H_2_O, thioacetamide, 130 °C for 12 h	1:2	0.01–3.0	0.1	277	200	[51]
SnCl_4_·5H_2_O, thioacetamide, 120 °C for 6 h	1:5.4	0.01–2.5	1	198	500	[52]

However, SnS_2_ anodes still suffer from low‐first‐cycle Coulombic efficiency (51%), relatively low intrinsic electronic conductivity of 3.9 × 10^−7^ (Ω cm)^−1^, and loss of capacity during cycling (due to volume expansion up to 300%).^[^ ^10^, ^11^ ^]^ To overcome the aforementioned problems, researchers have synthesized composites of SnS_2_, in which the SnS_2_ is deposited on or incorporated into graphene oxide, graphene substrates, and other forms of carbonaceous materials, as these nanostructures can better release strain and stresses, buffer the volume changes of the alloying reaction, and enhance electronic conductivity.^[^ ^12^, ^13^, ^14^, ^15^, ^16^, ^17^, ^18^ ^]^ Since the mass production of graphene and graphene oxide is prohibitively expensive and complex, such SnS_2_‐based composite materials are still largely not considered for commercial LIB applications.^[^ ^19^, ^20^ ^]^

In this work, the hydrothermal method was used to synthesize SnS_2_ nano powders with different crystallinities. A detailed literature survey on the hydrothermal synthesis of SnS_2_ revealed that most of the studies performed electrochemical testing of the SnS_2_ products that were synthesized using a single ratio of precursors, see Table 1. In some cases, surfactants and different sulfur sources were used for the synthesis, the SnS_2_ electrodes had higher amounts of conductive carbon (>20 wt%), electrode processing was performed using PVDF (Polyvinylidene fluoride)‐binder and NMP (N‐methyl‐2‐pyrrolidone) solvents, and nonstandard electrolytes were employed for half‐cell testing. Furthermore, a systematic investigation of the influence of precursor ratio on the structure–property–performance relations for this class of anode‐active materials was not performed. However, an in‐depth understanding of the influence of crystallinity and phase purity on electrochemical cycling in different potential windows is required to improve the electrochemical performance. Therefore, in the present work, SnS_2_ materials were synthesized using 1:2, 1:4, and 1:6 molar ratios of tin (IV) chloride pentahydrate (SnCl_4_.5H_2_O) and thioacetamide (C_2_H_5_NS) precursors, respectively, see Figure 1. The presence of nanocrystalline domains and the level of crystallinity in the SnS_2_ nanopowders were examined by powder X‐Ray diffraction analysis (XRD) and transmission electron microscopy (TEM). Our results indicate that over‐stochiometric precursor conditions are required for improved crystallinity. We also processed the electrodes using aqueous binders, underpinning the environmental sustainability of SnS_2_‐based anodes. Moreover, 10 wt% conductive carbon and 80 wt% active material were used in our electrode formulations. Electrochemical testing was performed using conventional electrolytes (1 M LiPF_6_ in 1:1 vol ratio EC (ethylene carbonate)/DMC (dimethyl carbonate) with 10 wt% FEC (fluoroethylene carbonate) additive) in half‐cell configurations. Although agglomeration of the particles occurred during electrode processing, different electrochemical performances properties for the SnS_2_ electrodes were obtained, indicating that the crystallinity of the as‐synthesized powders has a predominant effect on electrochemical performance, and this influence supersedes that of the electrode processing. In situ electrochemical impedance spectroscopy (EIS) and galvanostatic intermittent titration technique (GITT) techniques were also performed to evaluate lithium diffusion coefficients (D_Li_^+^) and understand the lithium intercalation kinetics of SnS_2_.

## 2 Results and Discussions

**Figure 2:**
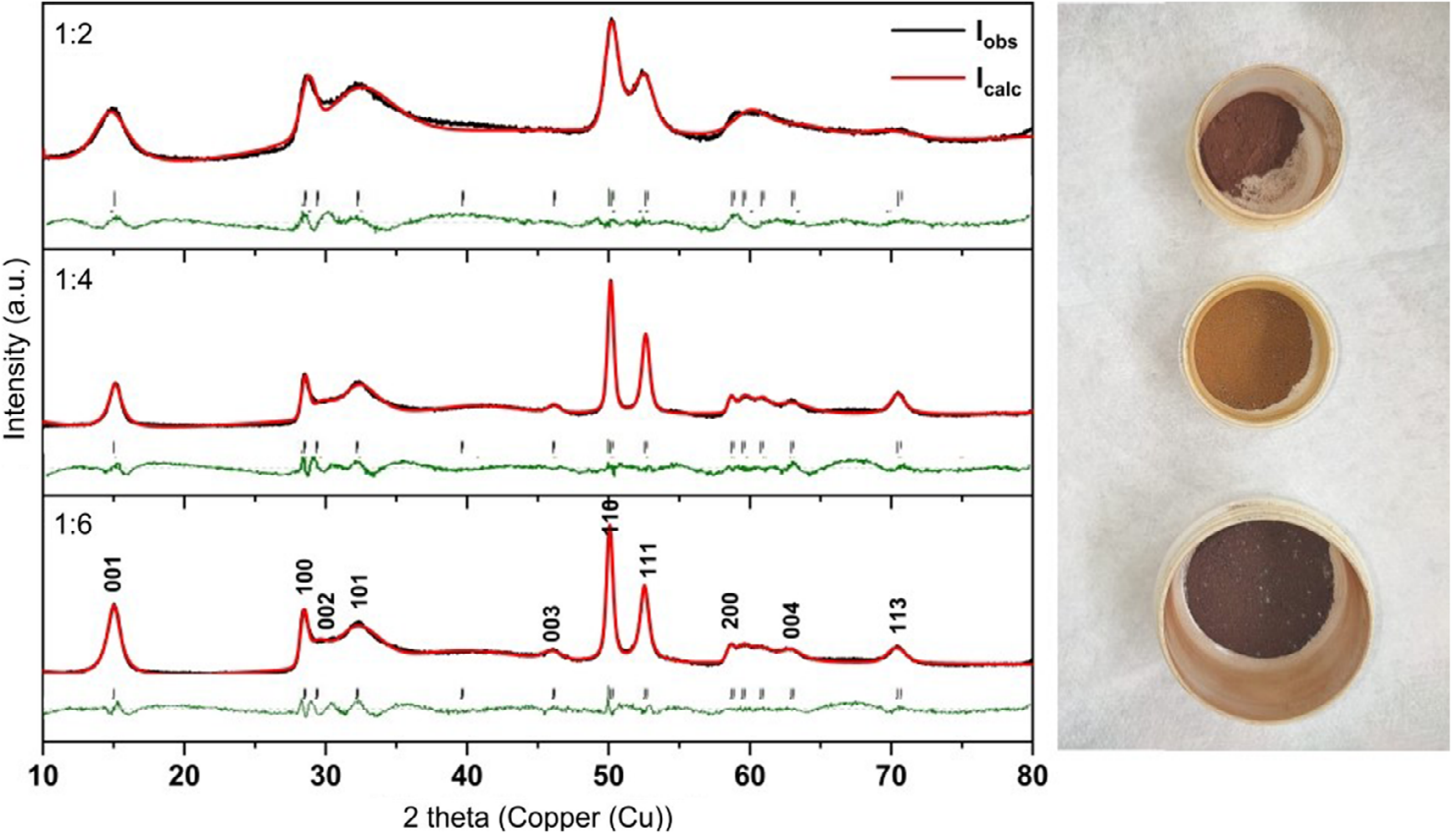
Powder XRD patterns of synthesized SnS_2_ powders for the samples (1:2, 1:4, and 1:6) as well their photo images from top to the bottom, respectively.

The crystal structure and phase purity of the “1:2”, “1:4,” and “1:6” SnS_2_ powders were characterized by powder XRD performed at room temperature. In the measured diffraction patterns shown in **Figure** **2**, crystalline SnS_2_ with space group P3m1 was identified. The intensities of the reflexes associated with the [110] and [001] planes differ for the samples, and the intensity ratios, determined as the quotient of the intensity of the [110] and the [001] peaks (*I*_[110]_/*I*_[001]_) for the “1:2”, “1:4,” and “1:6” samples were determined to be 2.3, 3.2, and 1.9, respectively. The diffractogram patterns exhibit sharper reflections and higher intensities for the 1:4 and 1:6 samples, whereas the XRD pattern of the 1:2 sample was characterized by broadened diffraction peaks with lower intensities and a higher background than in the other samples. In addition, the reflexes for the [003] and [113] crystal planes are not observed for the 1:2 sample, but they are present in the measured XRD patterns of the 1:4 and 1:6 samples. These effects are associated with the lower crystallinity of the 1:2 sample, as well as the change in crystallite sizes. The unit cell parameters and crystallite sizes of the samples determined using Rietveld refinement of the measured XRD patterns are compiled in **Table** **2** (see also Figure 2). The results of the Rietveld refinement indicate crystallite sizes of ≈5 and 9.2 nm for the 1:2 and 1:4 samples, respectively, whereas that for the 1:6 sample was 7.8 nm.

**Table 2:** Results of the Rietveld refinement of the SnS_2_ samples.

Sample	Lattice parameters [Å]	Crystallite sizes [nm]	*R*_p_	*R*_wp_
1:2	*a* = 3.625, *c* = 5.974	5	3.6	4.8
1:4	*a* = 3.622, *c* = 5.927	9.2	3.9	5.6
1:6	*a* = 3.617, *c* = 5.899	7.8	3	3.9

**Figure 3:**
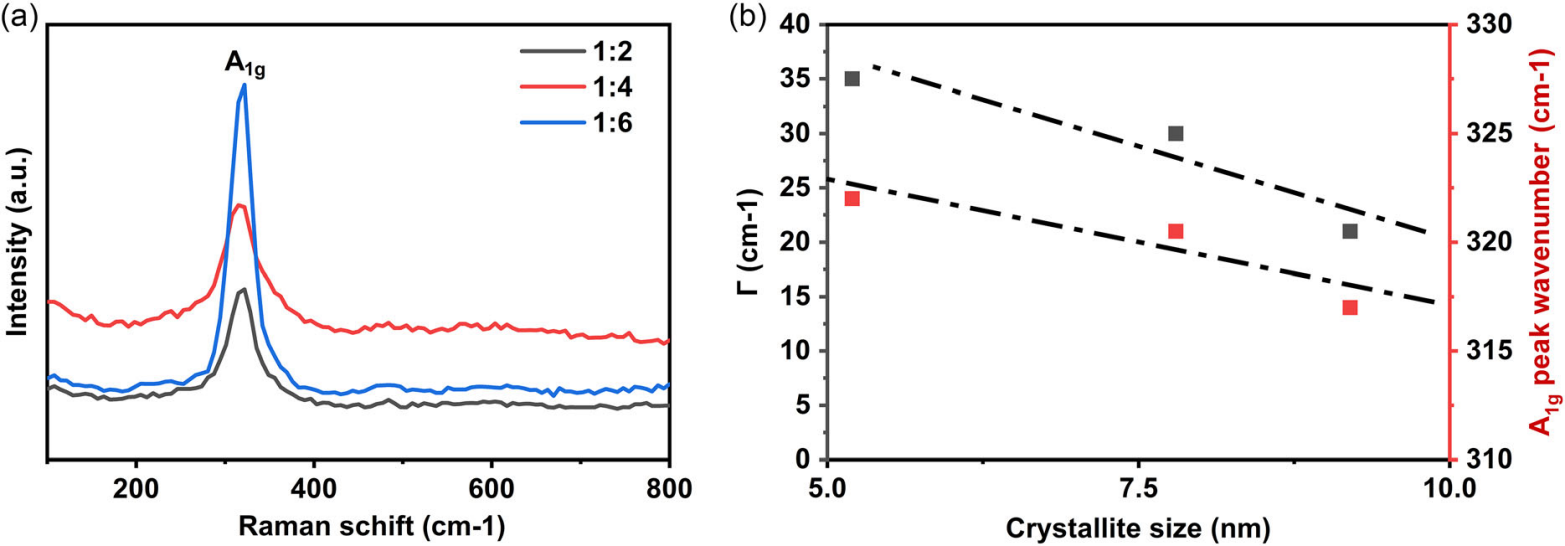
a) Raman Spectra of the synthesized SnS_2_ powders and b) full width at half maximum (Γ) and peak shift versus crystallite sizes.

Raman spectroscopy data exhibited a visible peak at about 317.8 cm^−1^ for the 1:4 sample, which corresponds to the A_1g_ mode of SnS_2_ due to the in‐plane vibrational modes of the sulfur–tin–sulfur bonds corresponding to compression of the SnS_2_ layers, see **Figure** **3**.^[^ ^21^, ^22^ ^]^ For the 1:6 and 1:2 samples, the A_1g_ peak position was observed at Raman shifts of 320.5 and 322 cm^−1^, respectively. The measured Raman peaks were fitted using Gaussian functions. In Figure 3b, the full width at half maximum (Γ) and the peak positions of the Gaussian fits are plotted against the crystallite sizes of the samples, which were obtained from the Rietveld refinements of the measured XRD patterns. Figure 3b shows that as the crystallite size increases, the Raman peak shifts toward lower wavenumbers. Furthermore, the full width at half maximum decreases as crystallite size increases. Frequently, polytypes of SnS_2_ demonstrate A_1g_ vibrational modes in the range 312–315.2 cm^−1^.^[^ ^21^, ^22^, ^23^ ^]^ Therefore, the Raman band broadenings and shifts observed for the 1:2, 1:4, and 1:6 samples were likely caused due to phonon confinement via crystal boundaries and defects, which in turn increases as the size of crystallite decreases. Similar trends are also observed in the literature.^[^ ^24^ ^]^ Another interesting observation is signal enhancement for the 1:6 sample. Similarly, resonance excitations have been observed in titanium dioxide semiconductors, where the effects of size and surface adsorption on surface‐enhanced Raman spectra and the phonon modes were studied.^[^ ^25^, ^26^ ^]^ The higher intensity of the A_1g_ peak for the 1:6 sample can originate from charge transfer resonance at the surface of the tin disulfide nanoparticles due to the presence of an adsorbed monolayer of molecules. These effects are further substantiated by the TEM investigations, which are detailed later in the manuscript.

**Figure 4:**
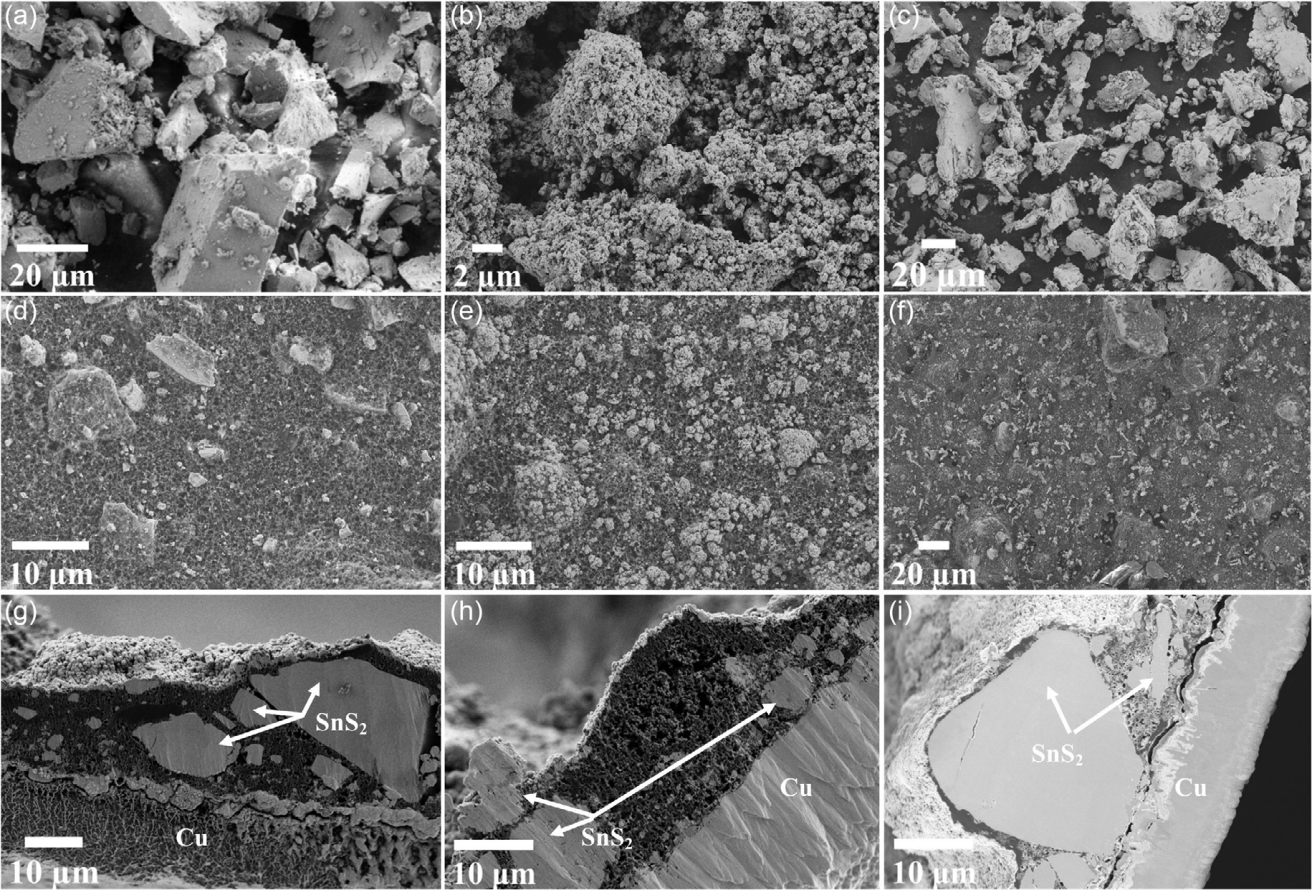
SEM images of SnS_2_ powders and pristine electrodes: a,d) for 1:2 sample, b,e) for 1:4 sample, and c,f) for 1:6 sample. Additionally, the cross‐section analysis of the pristine electrodes is shown: g) for 1:2, h) for 1:4 and i) for 1:6 samples.

The morphology of the three SnS_2_ samples is shown in **Figure** **4**. The secondary particles for the 1:2 and 1:6 samples were found to be irregularly shaped, with particle sizes in the range of 10–40 μm, as shown in Figure 4a,c. On the other hand, a smaller spherical morphology could be observed for the 1:4 particles, with an average particle size of 300 nm, Figure 4b. The electrode surfaces are shown in Figure 4d–f, whereas selected cross sections are displayed in Figure 4g–i. After electrode processing, agglomeration of the particles of the 1:4 sample has occurred, leading to larger secondary particles. According to the surface images, the dispersion of the particles is more homogeneous for the 1:4 sample electrode (e) than for (d and f) other two samples. However, the cross‐section images show nonhomogeneous layer thickness, common for all samples, since some of the agglomerated particles are larger than the dry thickness of the electrodes, leading to the presence of particle projections that are covered by the conductive additive. Despite the agglomeration, the active particles for the 1:4 samples are still smaller than those for the 1:2 and 1:6 electrodes. It can also be observed that the coatings well attached to copper current collector and the active materials are in close contact to conductive carbon matrix for all samples.

**Figure 5:**
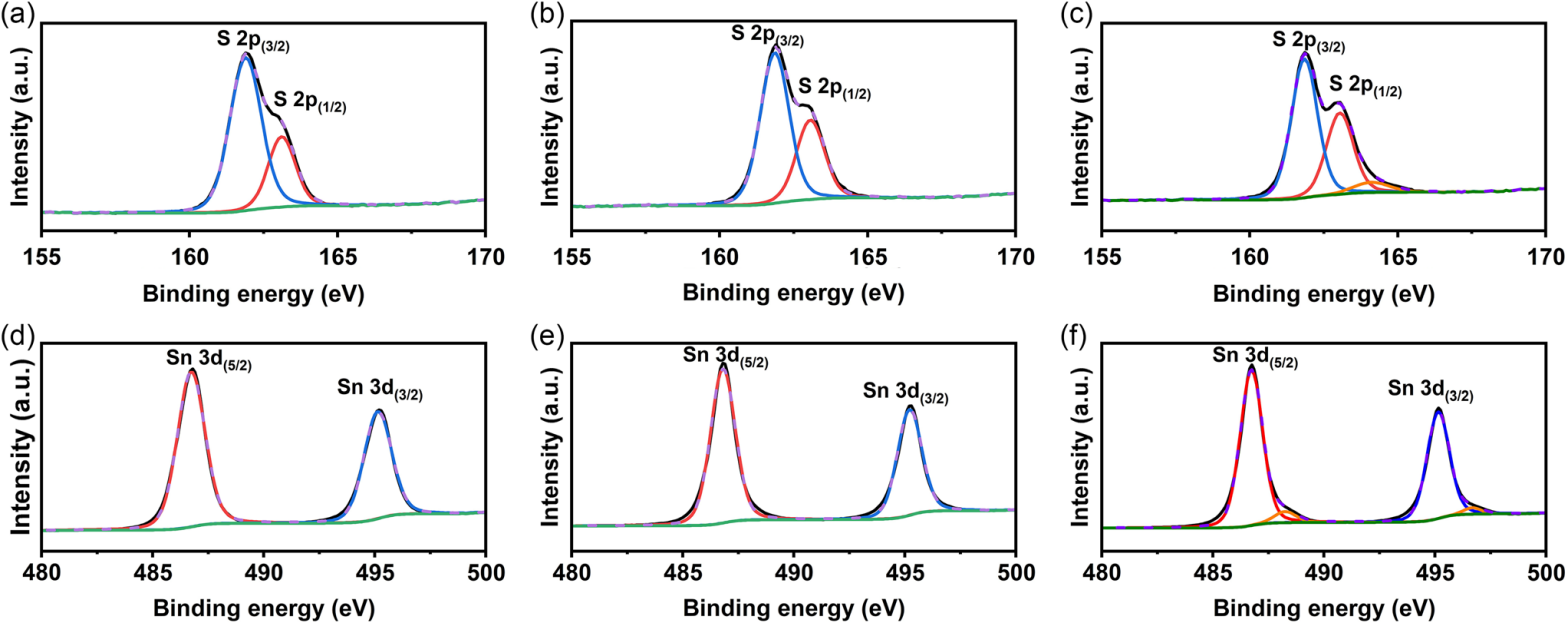
XPS spectra of S 2p and Sn 3d of SnS_2_ powders for a,d) 1:2, b,e) 1:4, and c,f) 1:6 samples.

X‐Ray photoelectron spectroscopy (XPS) measurements were performed to study the stoichiometric composition and oxidation states of Sn and S in the synthesized powders. **Figure** **5** shows the Sn 3d and S 2p regions, which were fitted by Voigt functions using the CASA XPS software. The energy calibration was performed based on referencing the C 1s peak to 284.6 eV, which corresponds to nonoxygenated carbon bonds. The binding energies of the S 2p peaks were fitted at 161.9 and 163.1 eV (see Table 2). This was observed for all samples and is ascribed to binding energies of the S 2p3/2 and 2p1/2 electron in S^2−^. The binding energies at 486.7 and 495.1 eV are assigned to the Sn 3d5/2 and Sn 3d3/2 electrons of Sn^4+^ in SnS_2_. The energy gap difference for the Sn^4+^ peaks is 8.4 eV, whereas that for the S^2−^ peaks is 1.2 eV, both of which are consistent with the literature.^[^ ^14^, ^23^ ^]^ In the Sn 3 d region of the 1:6 sample, additional peaks could be observed at 496.84 eV and 488.4 eV, which could originate from the presence of tin as a metal chelate formed from the starting materials.^[^ ^27^ ^]^ Similarly, at a high concentration of thioacetamide, a small peak at 164.1 eV was observed, which is presumably associated with intermediate byproducts of the main reaction, such as thiophene.^[^ ^28^ ^]^ According to the stochiometric atomic concentrations (see **Table** **3**), which were calculated by area integration analysis of the XPS data, the 1:2 sample contained significantly less sulfur that that expected for the stoichiometric compound, the 1:4 sample was close to the expected SnS_2_ stoichiometry, whereas the 1:6 sample exhibited an excess of S. Table 3 also shows the calculated S/Sn ratios as obtained from the evaluation of the XPS data. As the ratio of the S to Sn precursor increases, the S/Sn ratio in the final samples also increases, going from the ideal 1.5 value for the 1:2 samples to an overstoichiometry of S in the 1:6 sample, which has a S/Sn ratio of 2.1.

**Table 3:** Binding energies of the XPS spectra peaks of Sn 3d, S 2p and their calculated atomic concentration analysis and S/Sn ratios.

Samples	S 2p_(3/2)_	S 2p_(1/2)_	Sn 3d_(5/2)_	Sn 3d_(3/2)_	C 1s	S [atom. %]	Sn [atom. %]	S/Sn ratio
1:2	161.9	163.1	486.7	495.1	284.6	60	40	1.5
1:4	161.9	163.1	486.8	495.2	284.6	65.36	34.64	1.9
1:6	161.9	163.1	486.7	495.1	284.6	67.8	32.2	2.1

**Figure 6:**
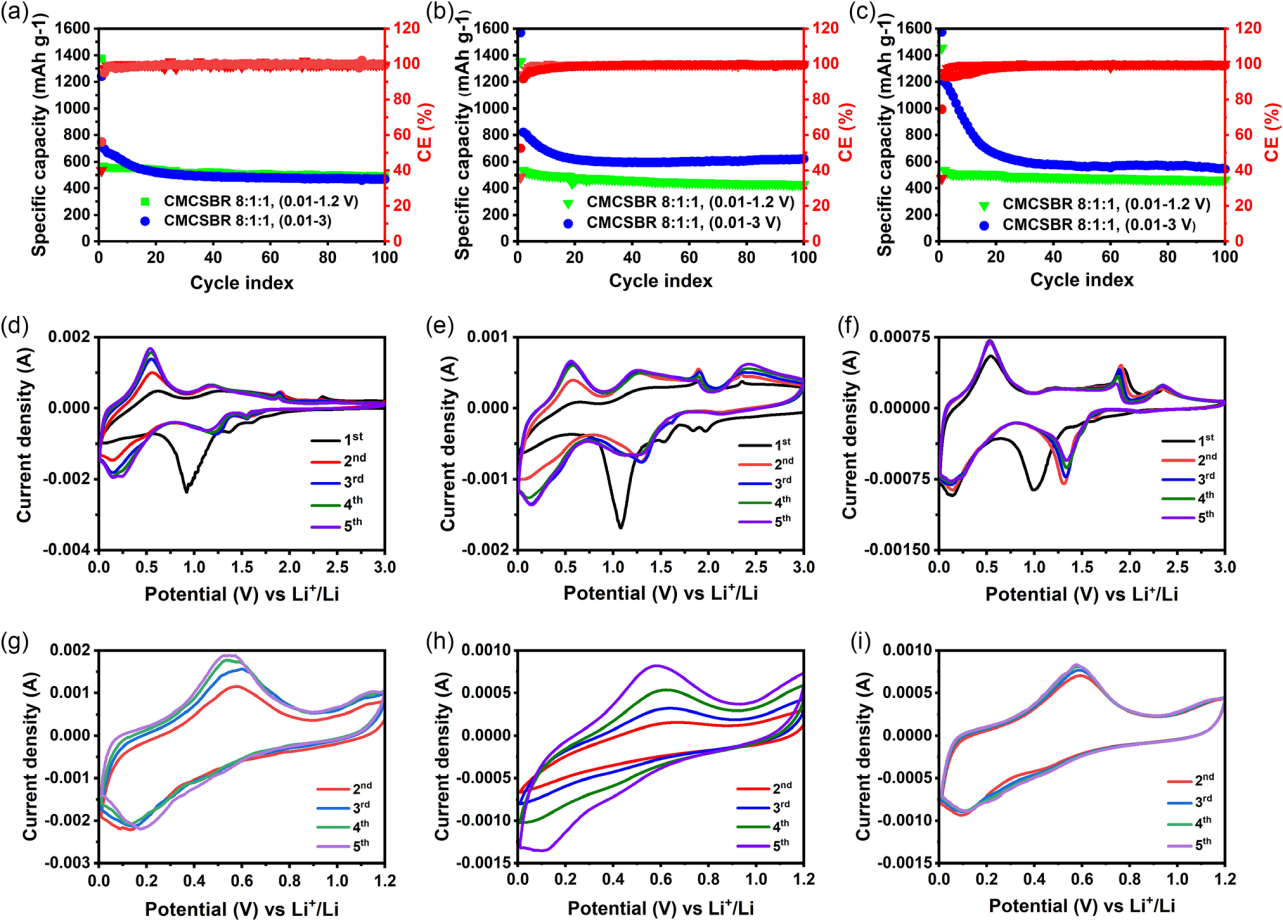
Cycling performance of SnS_2_ electrodes prepared with CMC–SBR water‐soluble binders. Comparatively, cycling potential windows were taken between 0.01 and 3 V and 0.01 and 1.2 V for each of the following samples: a) 1:2, b) 1:4, and c) 1:6. CV of SnS_2_ electrodes at scan rate 0.2 mV s^−1^ in potential admittances of 0.01–3.0 V and 0.01–1.2 V shown d,g) for 1:2, e,h) for 1:4, and f,i) for 1:6 samples, respectively.

The results of the electrochemical cycling performance of the three samples at 0.1 C rate (123 mA g^−1^, based on a theoretical specific capacity of 1230 mAh g^−1^) are shown in **Figure** **6**a–c for the 1:2, 1:4, and 1:6 samples, respectively. The initial CEs and retained specific capacities after 100 cycles are given in **Table** **4**. For these experiments, the lithiation step in the first cycle was performed at a slower specific current of 60 mAh g^−1^ to aid in the formation of a stable solid–electrolyte interphase (SEI) at the active material/electrolyte interface. All three samples were tested with a constant current constant voltage protocol with the termination of the constant voltage step at a current cutoff of 0.05 C.

**Table 4:** Initial CE and capacity retention after 100 cycles for the electrodes prepared from the 1:2, 1:4, and 1:6 samples in the 0.01–3 V and 0.01–1.2 V potential windows.

Samples	Initial CE [%]		Capacity retention after 100 cycles [mAh g^−1^]	
	0.01–3.0 V	0.01–1.2 V	0.01–3.0 V	0.01–1.2 V
1:2	57	39	487	462
1:4	52	36	598	428
1:6	71	35	544	462

In Figure 6a, electrodes from the 1:2 sample showed similar specific capacities in the 0.01–1.2 V and 0.01–3 V versus Li/Li^+^ voltage windows after the 20^th^ cycle. At the 100^th^ cycle, the retained capacities were 487 and 467 mAh g^−1^, respectively. At the end of 100 cycles in the voltage window between 0.01 and 3.0 V versus Li/Li^+^, electrodes from the 1:4 and 1:6 samples exhibited reversible capacities of 598 and 544 mAh g^−1^, respectively, with 99% CEs. However, after cycling in the voltage window of 0.01–1.2 V versus Li/Li^+^, electrodes prepared from the 1:4 and 1:6 SnS_2_ samples demonstrated capacities of 428 and 462 mAh g^−1^, respectively. The initial CE for the samples 1:2, 1:4, and 1:6 was found to be 39, 36, 35% in 0.01–1.2 V and 57, 52, 74% in 0.01–3 V potential ranges, respectively. In all the samples, there is a significant decrease in capacity in the first 20 charge/discharge cycles when cycling is performed between 0.01 and 3 V versus Li/Li^+^. This phenomenon was not observed when an upper cut‐off voltage of 1.2 V versus Li/Li^+^ was used for the cycling protocol.

To investigate these effects further, cyclic voltammetry (CV) measurements were performed on all samples in the full potential window of 0.01–3 V versus Li/Li^+^. The results of these experiments are shown for five cycles in Figure 6d–f. In all cases, a broad reduction peak with a minimum at ≈1 V versus Li/Li^+^ was observed in the first discharge. This is associated with the formation of Li_2_S and Sn according to the conversion reaction as shown in Equation (2). The large peak is followed by a broader reduction effect between 0.5 and 0.1 V versus Li/Li^+^, which is attributed to the alloying reactions of Sn with Li to form the Li_*x*_Sn alloy phases as indicated in Equation (3). Interestingly, the cathodic peaks that should be associated with the intercalation of lithium into the SnS_2_ crystal structure, as commonly observed in the literature,^[^ ^17^, ^29^ ^]^ differs significantly across the samples that were investigated in this work. For electrodes from the 1:2 sample, a broad peak at 1.9 V versus Li/Li^+^ was followed by two reduction peaks with minima at 1.6 and 1.35 V versus Li/Li^+^, respectively. For the 1:4 sample, distinct reduction peaks were measured at 1.95, 1.85, and 1.55 V versus Li/Li^+^, which were followed by a broad reduction peak at 1.0 V versus Li/Li^+^. For the 1:6 sample, on the other hand, one broad reduction peak with small offsets at 1.9, 1.62, and 1.4 V were observed. In all cases, however, the reduction peak for the conversion reaction in subsequent discharge cycles shifts to a higher potential of 1.35 V versus Li/Li^+^, which is preceded by a small peak at 1.55 V versus Li/Li^+^. However, this peak becomes less prominent and broader as the precursor ratio increases from 1:2 to 1:6.

In the oxidation scan, two effects are observed in the low‐potential range at ≈0.1 and 0.5 V versus Li/Li^+^, respectively. These are assigned to the dealloying reactions of Li_*x*_Sn and are followed by a peak at 1.9 V versus Li/Li^+^, which is potentially related to the formation of SnS_2_. The oxidation peaks at 2.3 V versus Li/Li^+^ may be due to the formation of sulfur or polysulfides,^[^ ^18^, ^29^, ^30^ ^]^ although these reactions were not further explored in this work. As the cycle number increases, the prominence of the oxidation peaks at the potentials larger than 1.5 V versus Li/Li^+^ decreases. This indicates that, as the cycle number increases, the extent of this reverse reaction decreases and the portion of the capacity originating from the alloying reaction increases. This is in accordance with the cycle performance of the electrode samples as observed in Figure 6a–c which showed a strong decrease in capacity during the first 20 cycles when the electrodes were cycled between 0.01 and 3 V versus Li/Li^+^, which is associated with the loss of capacity in the high‐voltage region above 1.5 V versus Li/Li^+^.

CV scans were also performed for the three samples in the voltage range from 0.01 to 1.2 V, and Figure 6g–i shows the data of the CV measurements starting from the second cycle. The results indicate that the redox reactions for the 1:6 sample and 1:2 samples are reversible within this potential window. However, the 1:4 sample shows poor reversibility of the oxidation and reduction peaks within the first five CV cycles. These results are in agreement with the cyclic performance of the electrodes in this potential window (see Table 4), as the electrodes from the 1:6 and 1:2 samples exhibit the highest retained capacities after 100 cycles (462 mAh g^−1^), whereas the 1:4 samples show a retained capacity of 428 mAhg^−1^, which is lower than that for the other two samples.

**Figure 7:**
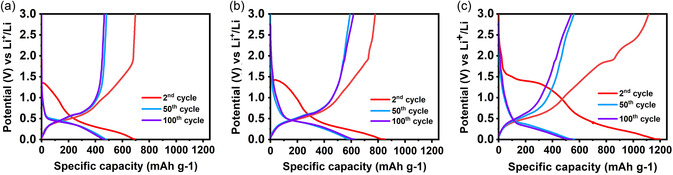
Galvanostatic charge and discharge curves of a) 1:2, b) 1:4, and c) 1:6 samples measured in the voltage range of 0.01–3.0 V were plotted for the 2^nd^, 50^th^, and 100^th^ cycles.

**Figure** **7**a–c shows the galvanostatic charge/discharge curves of 2^nd^, 50^th^, and 100^th^ cycles. The plateau for the conversion reaction during the second discharge is significant for the 1:6 sample at 1.3 V but less prominent for the 1:2 and 1:4 samples. This indicates some measure of reversibility of the conversion reaction for the 1:6 sample on the first charge cycle.

Postmortem scanning electron microscopy (SEM) analyses of the electrodes were performed for the three samples after 100 cycles in the potential window (0.01–3 V). The SEM images are shown in Figure S1a–c, Supporting Information and the micrographs indicate that, due to cycling, agglomeration of the particles took place, and cracks were observed for all samples.

**Figure 8:**
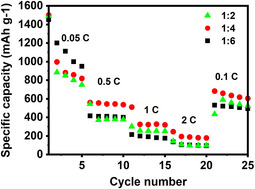
Comparative rate capability performances of SnS_2_ samples in 0.01–3.0 V range are shown.

To evaluate the rate capability of the electrodes, current densities of 0.05, 0.5, 1, 2, 0.1 A g^−1^ were applied to the cells on both the charge and discharge cycles, and the data are shown in **Figure** **8**. The best performance was observed for the 1:4 sample, which delivered capacities of 818, 509, 320, 178, and 605 mAh g^−1^ at the investigated current rates, respectively. At a current density 0.5 A g^−1^, the 1:2 and 1:6 samples show significant capacities of 371 and 405 mAh g^−1^, accordingly. However, when a current density of 2 A g^−1^ was applied, the capacities of the latter two samples reached 98 mAh g^−1^, which is only 45% of the capacity of the electrodes from the 1:4 samples when cycled at the same rate.

**Figure 9:**
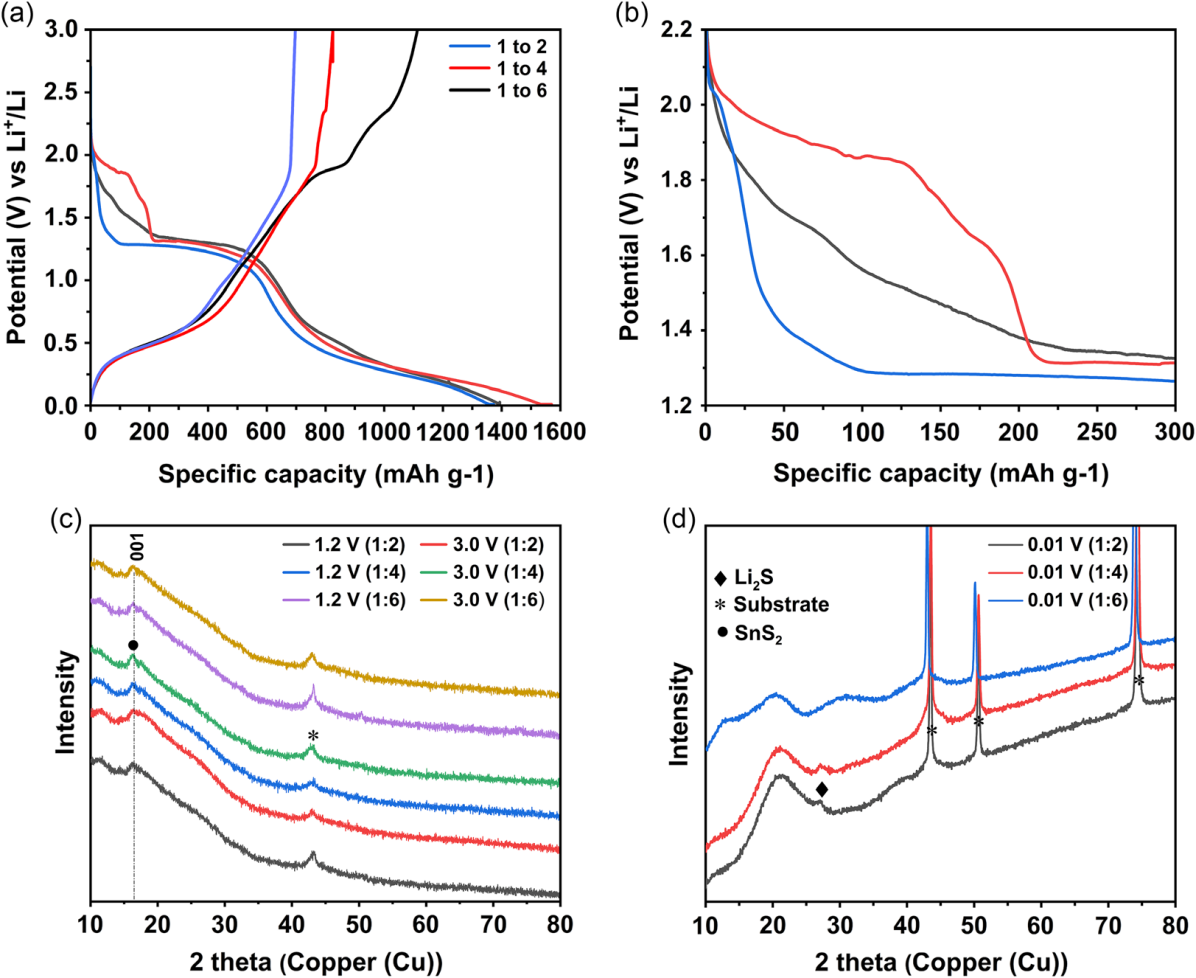
Postmortem XRD analysis of 1:2, 1:4, and 1:6 electrodes at 1.2 V and 3.0 V at c) first charged and 0.01 V d) discharged states, respectively. a) First discharge/charge capacity plateau versus potential and b) its initial *x* ≈ 2.08 or Li_*x*_SnS_2_ discharge profile.

The voltage profiles of the first discharge and charge cycles of the samples are shown in **Figure** **9**a. The first‐cycle discharge capacities were measured at 1348, 1529, and 1391 mAh g^−1^ for the 1:2, 1:4, and 1:6 samples, respectively. Since the theoretical capacity of SnS_2_ is 1230 mAh g^−1^, the higher discharge capacities may be due to formation of the SEI in the first discharge cycle. The large plateau at 1.3 V versus Li/Li^+^, which was observed in all samples, is associated with the conversion to metallic Sn and Li_2_S. This potential corresponds to the onset of the reduction peak that is associated with the conversion reaction in the CV scan, which showed a maximum current density at 1.0 V versus Li/Li^+^. The change in slope slightly below 0.5 V versus Li/Li^+^ on discharge corresponds to the reduction peak at similar potentials in the CV scan, whereas the change in slope of the charge curves at ≈0.3 and 0.5 versus Li/Li^+^ corresponds to the two, low‐potential oxidation peaks in the CV scans at ≈0.1 and 0.5 V versus Li/Li^+^, respectively. During charging, further changes in slope were detected at 1.9 and 2.3 V versus Li/Li^+^. These changes were most prominent for the electrodes from the 1:6 sample and are in good agreement with CV curves of that sample, where oxidation peaks were observed at 1.9 and 2.3 V versus Li/Li^+^. In all other samples, the oxidation peak at 2.0 V versus Li/Li^+^ can be correlated to the change in slope of the charge curve at 1.9 V versus Li/Li^+^.

To investigate the phase formation and development in the first discharge and charge cycle, ex situ XRD was performed in transmission mode on the electrodes discharged to 0.01 V versus Li/Li^+^ (Figure 9d) and in reflection mode on the electrodes charged up to 1.2 and 3.0 V versus Li/Li^+^ (Figure 9c). In Figure 9d, the Li_2_S phase was identified as a broad peak at 2θ = 27° only in discharged electrodes for the 1:2 and 1:4 samples. This peak is attributed to the [111] plane in the Li_2_S structure. Few authors reported about reversible formation of SnS_2_ from oxidation of Sn and Li_2_S.^[^ ^31^, ^32^ ^]^ In the charged state, the measured XRD patterns exhibited weak reflections at 2θ = 16°, which can be associated with the [001] crystal plane of the 164‐space group. This can be explained by the presence of residual SnS_2_, which did not completely react with Li at the lower potentials on discharge and is retained at 1.2 and 3.0 V on charge. Interestingly, no peaks were observed which could be used to potentially identify the presence of Sn in the charged state of the electrodes. Thus, the above results indicate, though not conclusively, the formation of Li_2_S after the conversion reaction on discharge and presence of nonreacted SnS_2_ during the first charge cycle. However, other techniques such as in situ TEM would be required for further confirmation.

During initial discharge, differences were observed in the voltage profiles measured up to 220 mAh g^−1^ or *x* ≈ 1.54 for all samples, see Figure 9d. This may be related to the lithiation mechanisms of the SnS_2_ particles with different crystallinity. First‐principal calculations have been widely performed in an attempt to understand the Li insertion and diffusion mechanisms into the SnS_2_ lattice structure. Liu et al. reported two steps for the lithiation process. In the first step, where *x* ≤ 1 in Li_*x*_SnS_2_, S atoms capture electrons from Li atoms, which are intercalated into the crystal lattice. In the second stage (1 ≤ *x* ≤ 3), Sn^4+^ cations are reduced to Sn and Li_2_S phase formation starts.^[^ ^33^ ^]^ Hassan et al. used computational modeling tools to suggest additional lithium storage between the Li−Sn and Li_2_S phase boundaries via an interfacial storage mechanism. According to their calculations, the interfaces contribute to charge storage after the alloying reaction, so that the total capacity can exceed theoretical values.^[^ ^34^ ^]^ Based on Sn Mossbauer spectroscopy, Lefebvre et al. reported that the intercalation of lithium ions up to (*x* ≈ 0.16) occurs via occupation of the vacant octahedral sites between layers, whereas up to *x* ≈ 1.21 in Li_*x*_SnS_2_, lithium can be inserted into the intralayer sites without destruction of the host crystal structure.^[^ ^35^ ^]^ Hwang et al.^[^ ^36^ ^]^ also reports on the non‐destructive intercalation of lithium up to *x* ≈ 1.0. Using in situ TEM, they showed that at higher lithium contents, a disordering of the crystal lattice can occur due to cation mixing of Li and Sn on the octahedral sites. This limits further intercalation of lithium and triggers the onset of the conversion reaction. Similar to Hwang et al.^[^ ^36^ ^]^ Gao et al. showed that each SnS_2_ unit cell can intercalate lithium up to *x* ≈ 1.0 with retention of the crystal lattice.^[^ ^37^ ^]^ Based on the experimental observations, few authors have tried to calculate the equilibrium voltage profile on first discharge of SnS_2_ to show the intercalation of Li in Li_*x*_SnS_2_ and formation of the Li_2_S phase, which results in a voltage plateau.^[^ ^31^, ^38^, ^39^ ^]^ However, the limits of lithium incorporation, that is, the value of *x* in Li_*x*_SnS_2_, are different or not given. Therefore, in this work, GITT measurements were used as a tool to probe the kinetics of the lithiation reaction of SnS_2_ up to 300 mAh g^−1^. This method can also be used to assess the extent of polarization during the constant current steps, as long relaxation steps are applied after the short galvanostatic pulse.^[^ ^40^ ^]^

**Figure 10:**
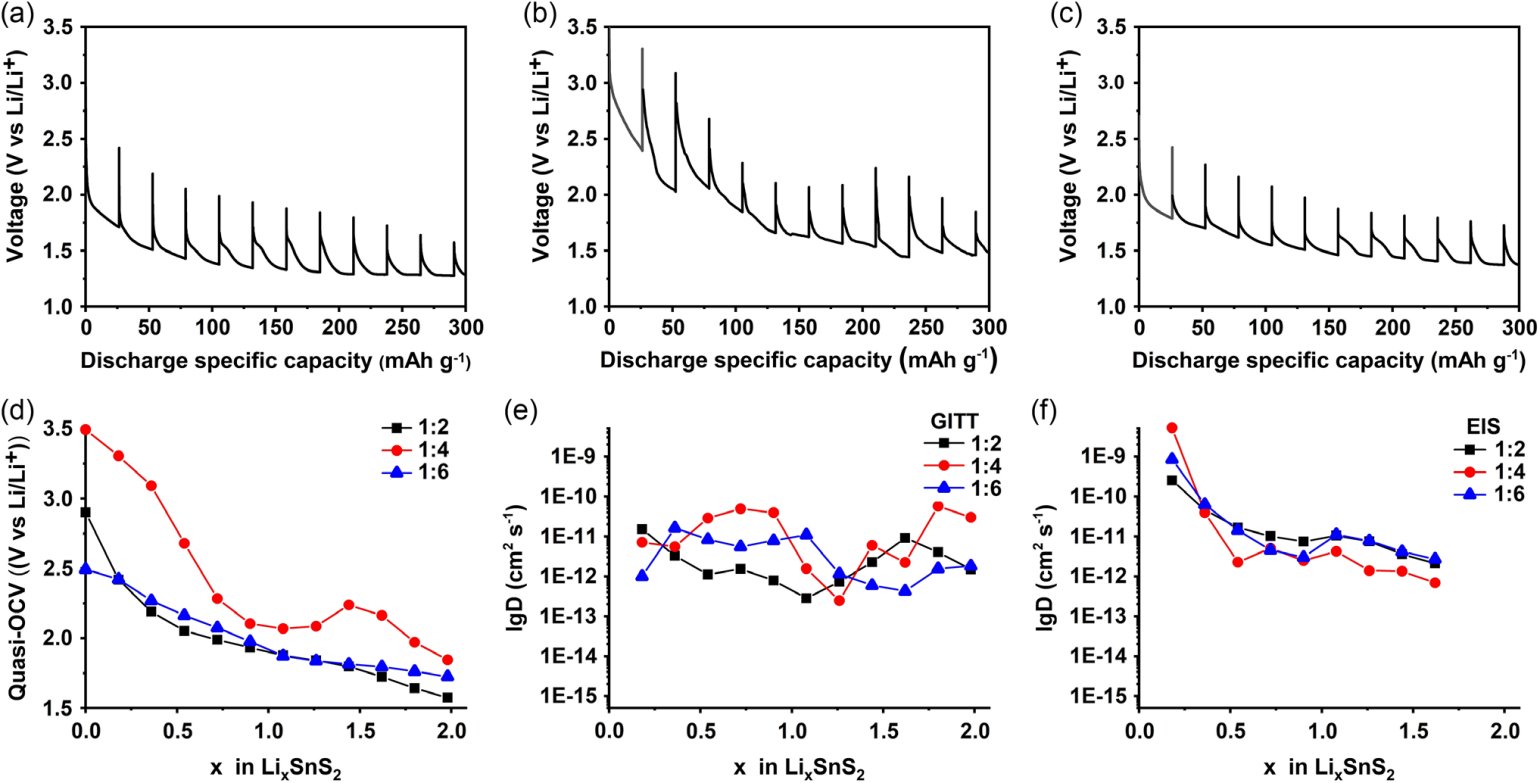
GITT of SnS_2_ composite electrodes at first discharge cycle from Li_*x*_ (*x* = 0) up to Li_*x*_ (*x* ≈ 2.08 or 300 mAh g^−1^) moles are shown, where short current pulses at C/23 rate value and 20 h relaxation time were programmed for a) 1:2, b) 1:4, and c) 1:6 samples. From calculated GITT of SnS_2_, lithiations were d) extracted via quasiequilibrium OCV pointsand e) in situ diffusion coefficients (D_Li_^+^) were calculated. f) Additionally, (D_Li_^+^) coefficients from in situ EIS technique were calculated and shown.

**Figure 11:**
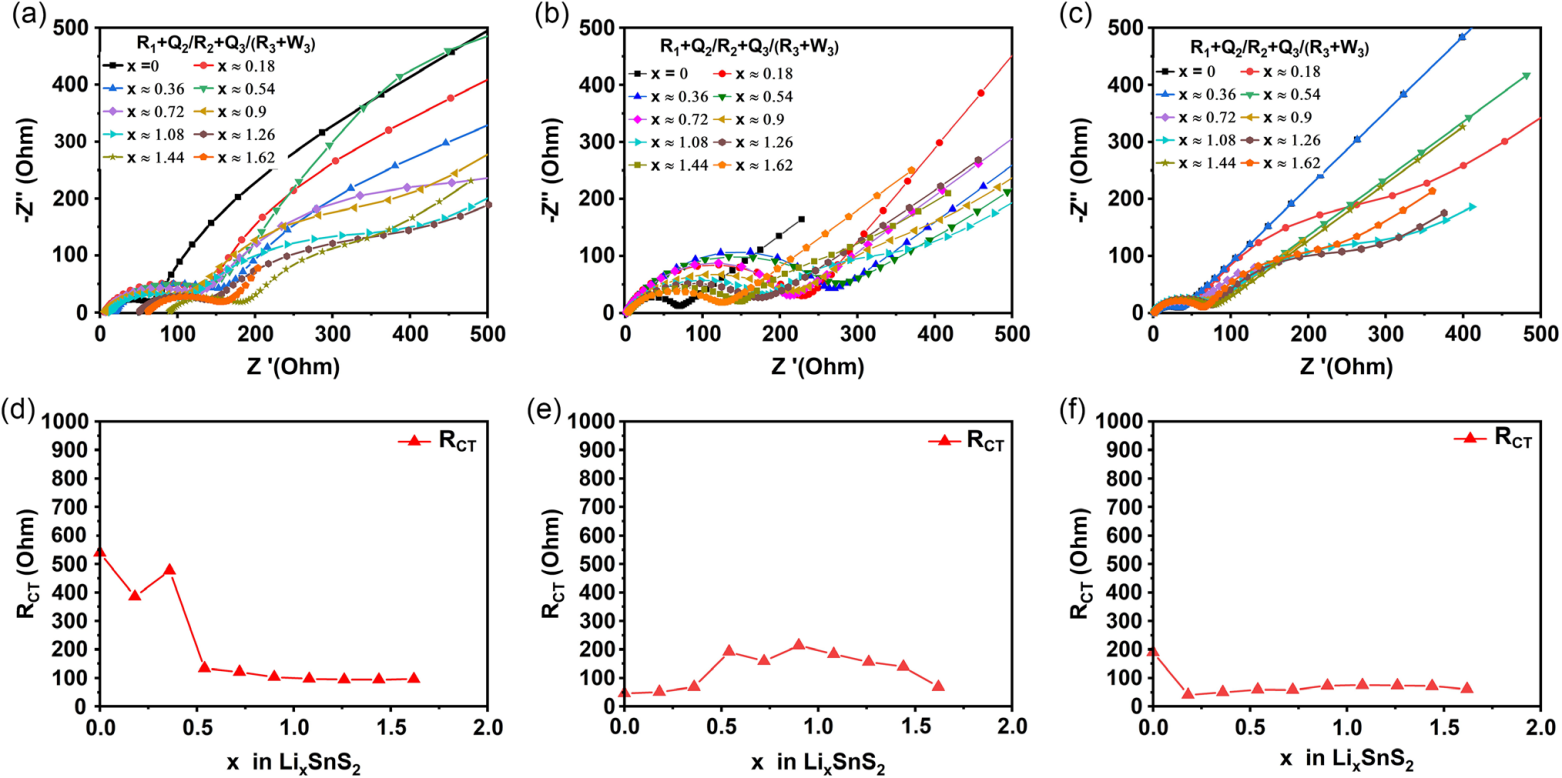
Fitted Nyquist Plots of in situ EIS of SnS_2_ at quasi‐equilibrium OCV points recorded after each *x* ≈ 0.18 (Li_*x*_SnS_2_) GITT lithiation step: a) 1:2 sample, b) 1:4 sample, and c) 1:6 sample. Obtained charge transfer resistance (R_CT_) and SEI resistance (R_SEI_) according to Table S2, Supporting Information: d) 1:2 sample, e) 1:4 sample, and f) 1:6 sample.

The GITT experiments were performed up to *x* ≈ 2.04 moles (294 mAh g^−1^) on fresh electrodes. Eleven current pulses at a C/23 rate (53.5 mA g^−1^) were applied for a duration of 30 min, with a 20 h relaxation time between pulses to cover the specific capacity range from 0 to 294 mAh g^−1^. The results of the measurements for electrodes prepared from the 1:2, 1:4, and 1:6 samples are shown in **Figure** **10**. The Li^+^ diffusion coefficients (D_Li_^+^) were calculated according to the derived Equation (4) of Weppner and Huggins.^[^ ^40^ ^]^

(4) $$\frac{dE}{d\surd t}\;=\;\frac{2I_{o}V_{\text{m }}}{SFz_{i}\surd \tilde{D}\pi }\;\frac{dE}{d\delta }\text{ or }\tilde{D}\;=\;\frac{4}{\pi }{\left(\frac{V_{\text{m }}}{SFz_{i}}\right)}^{2}{\left[\frac{I_{o}\left(\frac{dE}{d\delta }\right)}{\left(\frac{dE}{d\surd t}\right)}\right]}^{2}$$

where $I_{o}$ is the galvanostatic current, *S* is the area of the electrolyte–electrode interface, *F* is Faraday's constant, $\frac{dE}{d\delta }$ is the slope of the coulometric titration curve, which is found by plotting the steady‐state voltages, $V_{m}$ is the molar volume of the sample, $\frac{dE}{d\surd t}$ is voltage as a function of time *t* during which the current is applied, and $z_{i}$ is the charge number of species ⅈ. Furthermore, in order to compare the values of D_Li_^+^ coefficients and complement the study, EIS tests were applied after the GITT steps, see **Figure** **11** and Table S1, Supporting Information.^[^ ^41^ ^]^ Using such measurements, the diffusion coefficient of Li^+^ can be calculated as

(5) $$D_{{\text{Li}}^{+}}=\;\frac{R^{2}T^{2}}{2A^{2}n^{4}F^{4}C^{2}\sigma^{2}}$$

where *R* is the molar gas constant (8.314 J mol^−1^ K^−1^), *T* is absolute test temperature (298 K), *A* is the surface area of the electrode (1.766 cm^2^), *n* is the number of transferred electrons, *F* is the Faraday constant (96 500 C mol^−1^), *C* is the lithium‐ion concentration (1 mol l^−1^), and *σ* is the Warburg coefficient obtained from fitting. Fittings were performed using the equivalent circuit (R_1_ + Q_2_/R_2_ + Q_3_/(R_3_ + W_3_), where R_1_ is related to the combination of the resistances arising from the current collectors, separator, and the electrolyte resistance, R_2_ and Q_2_ refer to SEI layer resistance and capacitance, and R_3_ and Q_3_ refer to the charge transfer resistance and capacitance on electrode/electrolyte interface, while the Warburg element (W_3_) is associated with the diffusion of Li^+^ into bulk material.

The diffusion coefficients as calculated by the EIS and GITT methods are shown along with the quasi‐equilibrium open‐circuit voltage (OCV) values in Figure 10d–f, and the values are listed in Table S1, Supporting Information. In Figure 10d, the variation of the OCV during the lithium intercalation reaction for the three samples is shown. Whereas the OCV decreases continuously with increasing state of lithiation for the 1:2 and 1:6 samples, they initially decrease up to *x* ≈ 1.0 for the 1:4 sample, then increase up to a local maximum at *x* ≈ 1.5, and then decrease again. The local minimum in the quasiequilibrium voltage at *x* ≈ 1.0 corresponds to the shoulder in the discharge curve at a capacity of 140 mAh g^−1^ (see Figure 9b), whereas the local maximum at *x* ≈ 1.5 corresponds to the beginning of the voltage plateau associated with the onset of the conversion reaction at a capacity of 220 mAh g^−1^ (see Figure 9b).

In Figure 10e, the diffusion coefficients of lithium in the electrodes as calculated from the GITT measurements are presented. Despite the variations, which were larger for the 1:4 sample than for the 1:2 and 1:6 samples, the diffusion coefficients of lithium remained constant in a wide range between 1 × 10^−13^ and 1 × 10^−10^ cm^2^ s^−1^ between *x* ≈ 0.2 and 2.0. On the other hand, the diffusion coefficients of Li^+^ as calculated from the EIS measurements decrease continuously for all samples. However, it must be mentioned that the order of magnitude of the diffusion coefficients is the same for both measurements.

The Nyquist plot fittings from the EIS measurement are shown in Figure 11 and their values are listed in Table S2, Supporting Information. R_CT_ is initially high for 1:2 sample, but then decreases significantly after *x* ≈ 0.5, see Figure 11a,d, where R_CT_ values approximating 100 Ω were measured. In the case of the 1:4 sample, R_CT_ increased and then decreased throughout the full EIS spectrum, but never exceeded 200 Ω, see Figure 11b,e. For the 1:6 sample, the R_CT_ resistances were below <100 Ω throughout the lithiation stages, see Figure 11c,f, and were, in fact, the lowest for all samples measured.

**Figure 12:**
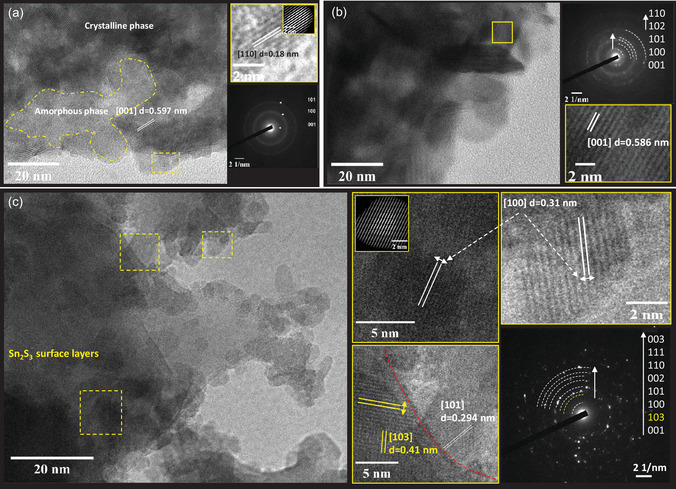
Morphology and structure characterization of the synthesized SnS_2_ powders using TEM and SAED. a) Distribution of SnS_2_ nanocrystals in amorphous matrix of the 1:2 sample, b) stack of crystallized SnS_2_ crystals for the sample 1:4, and c) dispersion of spherical‐shape 1:6 crystals trapped in between Sn_2_S_3_ layers, magnified and FFT images on chosen spots.

To further correlate the varied A_1g_ Raman resonance, the kinetics of lithium diffusion, as well as the variations in charge transfer resistances and first‐cycle discharge capacities with the nanostructure of the samples, ex situ TEM analysis was performed on the three powder samples, see **Figure** **12**. In all cases, small crystallites were observed to be dispersed within an amorphous matrix. These observations agree with the measured XRD patterns of the samples, which exhibit a high background due to the amorphous phase and broad diffraction peaks associated with the presence of small crystallites, see Figure 2. Figure 12a shows that the nanocrystalline regions of the 1:2 SnS_2_ sample formed platelets, with few layers (<7 layers) aligned along the *c*‐direction of the unit cell. The distance between the layers is *d* ≈ 0.597 nm for the [001] plane, which is in good agreement with the *c*‐parameter of the unit cell as determined by Rietveld analysis of the measured XRD pattern (*c* = 0.5974 nm). Furthermore, the formation of the [110] and [101] crystal planes could be observed, see inset. The selected‐area electron diffraction (SAED) pattern demonstrated the polycrystalline nature of the sample, with weak diffraction rings. However, bright spots associated with the [001], [100] and [110] planes of the crystalline, hexagonal SnS_2_ were also noticed in Figure 12a.

The 1:4 sample was also high polycrystalline, as shown in image Figure 12b. The SnS_2_ particles adopted a plate‐like morphology, where most of the plates aligned with the c‐axis ([001] direction). An interlayer *d*‐spacing of 0.586 nm was measured, which is slightly lower than the *c*‐axis lattice parameter determined by Rietveld refinement of the measured XRD pattern of the 1:4 sample (*c* = 0.5927 nm). The plate thickness varied from 5 nm to 15 nm, indicating a maximum of 22 unit cell layers. In the SAED pattern (Figure 12b), the 1:4 sample showed bright dots on the diffraction rings, where are associated with well‐crystallized planes of SnS_2_.

In the 1:6 sample, the SnS_2_ particles adopted a spherical shape morphology with sizes ranging between 5 and 10 nm (see Figure 12c). Interestingly, Sn_2_S_3_ layers were discovered on the surface of the 1:6 SnS_2_ crystallites. The SnS_2_ particles were trapped under these Sn_2_S_3_ layers, as the [101] and [100] planes of the 164‐space group were interfered by the [103] *d* ≈ 0.41 nm plane of the Sn_2_S_3_ 62‐space group (PDF 75‐2183). The SnS_2_ crystallites with [100] and [101] facets and *d* ≈ 0.31 nm and *d* ≈ 0.294 nm, respectively, were revealed on the cracked edge of grains. The SAED pattern in Figure 12c demonstrates the formation of all tin disulfide facets, and the [101] plane shows a bright atomic reflection. The Sn_2_S_3_ [103] plane ring was detected with low intensity, as shown in Figure 12c.

The TEM images reveal that the thickness of the Sn_2_S_3_ layers is below 10 Å. Therefore, no X‐Ray reflection peaks (see Figure 2) were observed in the measured XRD patterns of the 1:6 sample.^[^ ^42^ ^]^ Although the detection of the A_g_ band of Sn_2_S_3_ is difficult to identify in the Raman spectra due to its low intensity and possible overlapping with the A_1g_ band from SnS_2_,^[^ ^43^ ^]^ the presence of adsorbed Sn_2_S_3_ layers on the surface of SnS_2_ crystals could have contributed to the resonance effects (see Figure 3a) and enhanced intensities of the A_1g_ symmetry mode of SnS_2_,^[^ ^25^ ^]^ (see Figure 3a). Biswanath et al.^[^ ^44^ ^]^ showed a layer‐dependent variation of peak positions and relative intensities depending on Raman spectra for MoS_2_ (up to seven monolayers), which are in good agreement with our observation.

## 3 Conclusion

The above studies revealed that changing the precursor ratio for the hydrothermal synthesis of SnS_2_ influences the structure and morphology of SnS_2_. We conclude that partially crystalline, fully crystalline, and crystalline SnS_2_ with surface‐covered Sn_2_S_3_ were formed, depending on the precursor ratio. The electron diffraction analysis confirmed the morphology and degree of crystallinity of all samples plus the formation of Sn_2_S_3_ layers. The electrochemical tests showed that improved crystallinity of the particles positively affects the cycling performance of SnS_2_ when cycling is performed in the full voltage window between 0.01 and 3 V. Specifically, the best cycling and rate performance were observed for the crystalline 1:4 sample, with residual capacities of 598 mAh g^−1^ after 100 cycles and 605 mAh g^−1^ after rate capability testing. Lithiation mechanism and kinetics studies were performed using EIS and GITT to understand the influence of morphology, degree of crystallinity, presence of Sn_2_S_3_ layers on the OCV, lithium intercalation limits (Li_*x*_SnS_2_), and diffusion coefficients. The diffusion coefficient values by EIS and GITT were found to be in the same range and seem to be independent of the morphology of the samples investigated in this work. We expect that this detailed study on the influence of precursor ratio on structural and electrochemical properties will open new avenues for further exploration of this class of materials for rechargeable batteries and other applications.

## 4 Experimental Section

4.1

4.1.1

##### Materials

Tin (IV) chloride pentahydrate (SnCl_4_·5H_2_O) from Alfa Aesar (98% purity), thioacetamide (C_2_H_5_NS) from Alfa Aesar (99% purity), deionized (DI) water, ethanol, dimethyl carbonate, glass beakers, magnetic stirrers, Teflon‐lined stainless‐steel autoclave 125 mL, filter funnel, mortar and pestle blade coater, carboxymethyl cellulose (CMC) from Alfa Aesar, styrene butadiene rubber (SBR) from JSR Micro, carbon black C65 from Imerys, coin cells (CR2032), Li chip from PI‐KEM, 1M LiPF_6_ in 1:1 EC/DMC with 10 wt.% FEC (fluoroethylene carbonate) from SOLVIONIC, and conductive carbon tape were used.

##### Synthesis Procedure of SnS_2_

Tin (IV) chloride pentahydrate (SnCl_4_·5H_2_O) and thioacetamide (C_2_H_5_NS) were dissolved in DI water separately in glass beakers. The mixture of the SnCl_4_·5H_2_O and C_2_H_5_NS solutions was vigorously stirred for 10 min using a magnetic stirrer, before it was transferred into 125 mL Teflon‐lined stainless‐steel autoclave. The closed autoclave was heated at 160 °C in an oven for 10 h, after which it was cooled to room temperature (see Figure 1). The precipitated products were further separated by centrifugation at 2500 rpm for 5 min (from Malvern PanAnalytical) using a filter funnel. They were washed three times with distilled water to remove any residuals and finally dried in vacuum at 65°C overnight. During synthesis, 1:2, 1:4, and 1:6 mole ratios of tin chloride to thioacetamide were used. The concentration of the initial tin chloride solution was fixed by dissolving 3 g (8.5 mmol) of tin (IV) chloride in 20 mL of deionized water. For the 1:2 molar ratio, 1.28 g (17 mmol) of thioacetamide was dissolved in 40 mL of deionized water, whereas for the 1:4 and 1:6 molar ratios, 2.56 g (34 mmol) and 3.85 g (51.24 mmol) of thioacetamide were dissolved in 40 mL of water, respectively. The latter mole variations were identified in the text as “1:2”, “1:4,” and “1:6”, respectively.

##### Instrumentation and Characterization

Powder XRD patterns were measured using a X’Pert Pro and Empyrean PANalytical diffractometers using Cu Kα radiation (*λ* = 1.542 A) over 2θ range 10°–80°. Phase identification and lattice parameter determination were performed by Rietveld refinement of the measured diffractograms. XPS (Nexsa, Thermofisher) was employed to study the surface composition and oxidation states of the synthesized SnS_2_ samples using the following acquisition parameters: Al K Alpha source gun, spot size 400 μm, pass energy 50 eV, and energy step size 0.1 eV, etching with monoatomic Ar beam at 6000 eV, 30s. TEM (JEM 1400Plus, Jeol 120 kV) was used to investigate phases and crystal structure. Samples for the TEM investigations were initially dispersed in ethanol, after which each the solution was added dropwise to the cooper grid sample holder. Additionally, Raman spectra were measured using a Horiba Scientific (Jobin‐Yvon‐Horiba) with 532 nm laser wavelength. SEM (Carl Zeiss Supra 40 scanning electron microscope) was used to investigate morphology of powders and electrodes. Samples for the SEM investigations were prepared by attaching powder/electrodes on carbon conductive tape and postmortem electrodes were washed in dimethyl carbonate solution and dried. The cross‐section samples were prepared using an argon‐ion milling device.

##### Electrochemical Testing

The as‐synthesized powders of SnS_2_ were ground using a mortar and pestle prior to preparation of the electrodes. The ground SnS_2_ powder was used as the active material (80 wt.%) for electrode production, whereas a 1:1 mixture of carboxymethyl cellulose (CMC) from Alfa Aesar (5 wt.%) and styrene butadiene rubber (SBR) from JSR Micro (5 wt.%) was used as the water‐based binder. In addition, carbon black C65 (10 wt.%) from Imerys was used as the conductive additive. DI water was used as a solvent to prepare the slurries with 25 wt% solid content. The components were mixed and homogenized at 3000 rpm 5 min in Thinky mixer, and the obtained slurries were blade coated on copper foil using a wet thickness of 60 μm. The electrodes were then dried for 10 h at 60 °C in a vacuum oven. Electrodes were cut into 15 mm‐diameter disks and assembled into coin cells (CR2032) in an Ar‐filled glove box (H_2_O and O < 1 ppm) in half‐cell configuration (against 15 mm Li chip from PI‐KEM). Celgard 2325 was used as a separator, and 40 μl of 1M LiPF_6_ in EC/DMC with 10 wt.% FEC (fluoroethylene carbonate) from SOLVIONIC was used as the electrolyte. Galvanostatic discharge and charge were performed at 0.1 C rate calculated from theoretical capacity of SnS_2_ 1230 mAh g^−1^. Cycling was performed on a battery tester (Arbin) with cut off potentials of 0.01 V versus Li/Li^+^ for the discharge and 1.2 V and 3.0 V versus Li/Li^+^ for the charge step, respectively. A Bio Logic battery tester was also used for galvanostatic cycling as well as for CV, which was performed at a scan rate 0.2 mV s^−1^. GITT experiments were conducted up to Li_*x*_SnS_2_ with *x* ≈ 2.08. Current pulses at a C/23 rate for a duration of 30 min were used, with a 20 h relaxation time between pulses in order to achieve a quasi‐equilibrium state. EIS was programmed after each GITT step in the frequency range from 10 mHz to 100 kHz.

## Conflict of Interest

The authors declare no conflict of interest.

## Author Contributions

**Akzhan Bekzhanov**: conceptualization (lead); data curation (lead); formal analysis (lead); investigation (lead); methodology (lead); writing—original draft (lead); writing—review and editing (equal). **Nurgul Daniyeva**: investigation (supporting); writing—review and editing (supporting). **Qixiang Jiang**: investigation (supporting); writing—review and editing (supporting). **Yuri Surace**: investigation (supporting); writing—review and editing (supporting). **Freddy Kleitz**: supervision (equal); writing—original draft (supporting); writing—review and editing (equal). **Damian Cupid**: data curation (supporting); methodology (equal); project administration (lead); resources (lead); supervision (lead); writing—review and editing (equal).

## Supporting information

Supplementary Material

## Data Availability

The data that support the findings of this study are available from the corresponding author upon reasonable request.
