# Supplementary material for: Hydrothermally Synthesized SnS2 Anode Materials with Selectively Tuned Crystallinity
Source: Small Sci. 2024 Dec 23;5(5):2400516. doi: 10.1002/smsc.202400516 (PMC12087779; doi:10.1002/smsc.202400516)
Supplement: Supplementary file 1 — Supplementary Material [file SMSC-5-2400516-s001.pdf]

Supporting Information

Hydrothermally synthesized SnS_2 anode materials with selectively tuned crystallinity

Akzhan Bekzhanov[1,2,5], Nurgul Daniyeva[3], Qixiang Jiang[4], Yuri Surace[1], Freddy Kleitz[2,*], Damian Cupid[1,*]

[1] *Center for Transport Technologies, Austrian Institute of Technology GmbH, 1210 Vienna, Austria;*
[2] *Department of Functional Materials and Catalysis, Faculty of Chemistry, University of Vienna, Währinger Str. 42, 1090 Vienna, Austria;*
[3] *Core Facilities, Nazarbayev University, Astana, Kazakhstan;*
[4] *Institute of Material Chemistry, Faculty of Chemistry, University of Vienna, Wähinger Strasse 42, 1090, Vienna, Austria;*
[5] *Vienna Doctoral School in Chemistry (DoSChem), University of Vienna, Währinger Str. 42, 1090, Vienna, Austria;*

* Corresponding authors: freddy.kleitz@univie.ac.at (F.K.); Damian.Cupid@ait.ac.at (D. C.)

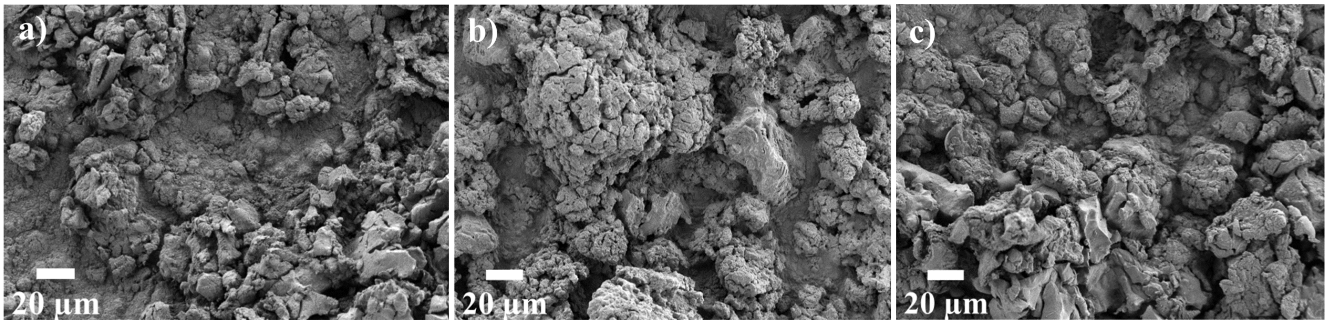

Figure S1. Pristine prepared composite electrodes (first row): a) for 1:2, b)) for 1:4 and c) for 1:6. Post-mortem surface micrograph analysis of cycled electrodes (second row) of d) 1:2, e) 1:4 and f) 1:6.

Table S1. The Li^+ Diffusion coefficients data calculated using in-operando EIS and GITT techniques up to 300 mAh g^{-1} point at first discharge cycle.

1:2				1:4				1:6			
Li_xSnS_2	OCV (V)	D_{Li} by EIS (cm^2 s^{-1})	D_{Li} by GITT (cm^2 s^{-1})	Li_xSnS_2	OCV (V)	D_{Li} by EIS (cm^2 s^{-1})	D_{Li} by GITT (cm^2 s^{-1})	Li_xSnS_2	OCV (V)	D_{Li} by EIS (cm^2 s^{-1})	D_{Li} by GITT (cm^2 s^{-1})
0	2.9				3.49				2.49		
0.18	2.41	2.51293E-10	1.52473E-11	0.18	3.30	5.26021E-9	7.07428E-12	0.18	2.42	8.47392E-10	1.00451E-12
0.36	2.19	4.58805E-11	3.29875E-12	0.36	3.09	3.90391E-11	5.48232E-12	0.36	2.26	6.3478E-11	1.62414E-11
0.54	2.05	1.66827E-11	1.10735E-12	0.54	2.67	2.25352E-12	2.85925E-11	0.54	2.16	1.42394E-11	8.32524E-12
0.72	1.98	1.0213E-11	1.53721E-12	0.72	2.28	5.03766E-12	4.93393E-11	0.72	2.07	4.5513E-12	5.49475E-12
0.9	1.93	7.39972E-12	7.86884E-13	0.9	2.10	2.47746E-12	3.95195E-11	0.9	1.97	3.01137E-12	7.87485E-12
1.08	1.87	1.04991E-11	2.80791E-13	1.08	2.06	4.22278E-12	1.54688E-12	1.08	1.87	1.11257E-11	1.09548E-11
1.26	1.84	7.5351E-12	7.3149E-13	1.26	2.08	1.37568E-12	2.46409E-13	1.26	1.83	7.70483E-12	1.15255E-12
1.44	1.79	3.60047E-12	2.25617E-12	1.44	2.23	1.33611E-12	5.92915E-12	1.44	1.81	4.17569E-12	5.95256E-13
1.62	1.72	2.09541E-12	9.09703E-12	1.62	2.16	6.89363E-13	2.24092E-12	1.62	1.79	2.7101E-12	4.26149E-13
1.8	1.64	--	4.03907E-12	1.8	1.96	--	5.72679E-11	1.8	1.76	--	1.55766E-12
1.98	1.57	--	1.49981E-12	1.98	1.84	--	3.00882E-11	1.98	1.72	--	1.82007E-12

Table S2. EIS fitting data for the Nyquist plot in Figure 9.

1:2			1:4			1:6		
**Li_xSnS_2**	**R_2/R_{SEI} Ohm**	**R_3/R_{CT} Ohm**	**Li_xSnS_2**	**R_2/R_{SEI} Ohm**	**R_3/R_{CT} Ohm**	**Li_xSnS_2**	**R_2/R_{SEI} Ohm**	**R_3/R_{CT} Ohm**
0	66	540	0	14	46	0	32.7	190
0.18	145	386	0.18	142	51	0.18	132	41
0.36	147	477	0.36	172	68	0.36	94	50
0.54	268	134	0.54	265	192	0.54	6.15	59
0.72	251	121	0.72	84	159	0.72	70	58
0.9	214	103	0.9	60	214	0.9	138	73
1.08	225	97	1.08	126	184	1.08	176	75
1.26	202	94	1.26	117	156	1.261	139	74
1.44	73	94	1.44	150	139	1.44	110	71
1.62	70	96	1.62	130	68	1.62	91	61
